# Correlation of Neuroimaging Findings with Clinical Presentation and Laboratory Data in Patients with COVID-19: A Single-Center Study

**DOI:** 10.1155/2021/2013371

**Published:** 2021-08-10

**Authors:** Seyhmus Kavak, Mehmet Serdar Yildirim, Rojhat Altındag, Yilmaz Mertsoy, Mehmet Fuat Alakus, Mehmet Diyaddin Guleken, Safak Kaya

**Affiliations:** ^1^Department of Radiology, Health Sciences University, Gazi Yasargil Research and Training Hospital, Diyarbakir, Turkey; ^2^Department of Internal Medicine, Health Sciences University, Gazi Yasargil Research and Training Hospital, Diyarbakir, Turkey; ^3^Department of Cardiology, Health Sciences University, Gazi Yasargil Research and Training Hospital, Diyarbakir, Turkey; ^4^Department of Orthopedics and Traumatology, Health Sciences University, Gazi Yasargil Research and Training Hospital, Diyarbakir, Turkey; ^5^Department of Ophthalmology, Health Sciences University, Gazi Yasargil Research and Training Hospital, Diyarbakir, Turkey; ^6^Department of Psychiatry, Health Sciences University, Gazi Yasargil Research and Training Hospital, Diyarbakir, Turkey; ^7^Depatment of Infectious Diseases and Clinical Microbiology, Health Sciences University, Gazi Yasargil Research and Training Hospital, Diyarbakir, Turkey

## Abstract

**Background:**

This study was aimed at revealing neuroimaging findings in COVID-19 patients and at discussing their relationship with epidemiological data and some laboratory parameters. *Materials and Method*. This study included 436 cases of COVID-19 and 40 cases of non-COVID-19 acute/subacute thromboembolism who underwent at least one neuroimaging procedure due to neurological symptoms between April 2020 and December 2020. The group of COVID-19-positive acute/subacute thromboembolism cases was compared with both the group of normal brain imaging cases and the non-COVID-19 acute/subacute thromboembolism group in terms of demographic data and laboratory parameters.

**Results:**

When the acute/subacute thromboembolism group and neuroimaging findings were compared in terms of negative group, presence of comorbid disease, D-dimer level, and lymphocyte count in COVID-19 patients, a statistically significant difference was found (*p* = 0.047, 0.014, and <0.001, respectively). COVID-19-positive and COVID-19-negative acute/subacute thromboembolism cases that were compared in terms of gender, neuroimaging reason, C-reactive protein, D-dimer level and lymphocyte count, a statistically significant difference was found (*p* = 0.003, <0.001, 0.005, 0.02, and <0.001, respectively).

**Conclusion:**

Acute thromboembolic events are common in patients with COVID-19 due to a potentially increased procoagulant process. Neurological evaluation and, if necessary, detailed neuroimaging should be performed, especially in cases with high D-dimer levels.

## 1. Introduction

After the novel coronavirus (nCoV) first appeared in Wuhan, China, in December 2019, it spread rapidly, causing a pandemic shortly thereafter. This new virus was named severe acute respiratory syndrome coronavirus 2 (SARS-CoV-2), and the disease it caused was named “coronavirus disease 2019” (COVID-19) [1, 2]. This virus exerts its effect by binding with high affinity to angiotensin-converting enzyme 2 (ACE2) receptors in humans via virus spike proteins. These receptors are located in many organs of the body, such as the brain. In the brain, ACE2 receptors are widely expressed in glial cells and brainstem nuclei involved in the regulation of the cardiorespiratory system, the reticular activation system, and the motor cortex [3]. It is controversial whether high ACE2 receptor density, especially at the nucleus solitarius and nucleus ambiguus levels, contributes to severe respiratory dysfunction [4].

Two hypotheses have been proposed regarding how the virus accesses the central nervous system (CNS): the first is hematogenous spread of infected leukocytes through the compromised endothelial cells of the blood–brain barrier, similar to the spread of other viruses, and the second is retrograde spread along the axons of peripheral nerves, such as the olfactory nerve [5]. Once the virus reaches the CNS, it causes damage either by the cytokine storm that occurs or by direct action. In addition, it has been indicated that increased coagulation activity and a correlated increase in D-dimer level may cause secondary damage and increase the risk of thromboembolism [6–9].

COVID-19 can present with neurological symptoms such as anosmia, headache, impaired taste sensation, dizziness, syncope, and altered consciousness [10]. It has been reported in previous studies that thromboembolic ischemic and hemorrhagic strokes associated with COVID-19 have been observed in the early period. It has been suggested that conditions characterized by neurological sequelae, such as encephalitis and encephalopathy, ataxic seizures, Guillain-Barré syndrome (GBS), demyelinating diseases, and neuromuscular disorders, may occur in the longer term [11–14].

The aim of this study is to discuss the relationship of brain imaging findings with laboratory and epidemiological data in patients with neurological symptoms followed up with the diagnosis of COVID-19. In addition, cases with acute/subacute thromboembolism without COVID-19 and cases with COVID-19-positive acute/subacute thromboembolism were compared.

## 2. Materials and Method

**Figure 1:**
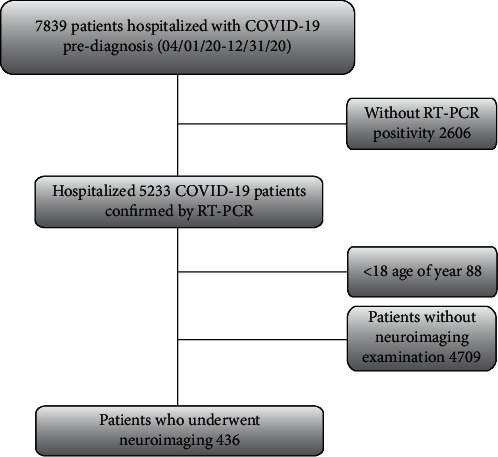
Flowchart of patient inclusion. COVID-19: coronavirus disease 2019; RT-PCR: reverse-transcription polymerase chain reaction.

We retrospectively analyzed 5233 real-time reverse transcriptase-polymerase-chain reaction- (RT-PCR-) positive cases hospitalized in our hospital with a diagnosis of COVID-19 between April and December 2020 and included 436 cases who underwent at least one neuroimaging (Figure 1). Computed tomography (CT) and magnetic resonance (MR) images of these patients were evaluated by 2 experienced radiologists (S.K. and R.D.). The opinion of a third radiologist (A.A.) was sought in cases of disagreement to achieve consensus. CT and MR images obtained during hospitalization or at the first admission to the hospital were examined for the presence of ischemia, infarction, bleeding, and encephalitis. The patient population was divided into 4 groups according to radiological data, and comparative statistical analyses of these groups were performed. The first group consisted of 46 patients with acute/subacute infarction with neuroimaging findings, and the second group consisted of the remaining 390 patients. Cases with nonspecific white matter changes on neuroimaging (*N* = 189) constituted the third group, and the remaining 247 cases constituted the fourth group. In addition, neuroimaging findings, clinical, epidemiological, and laboratory data of 40 patients diagnosed with non-COVID acute/subacute thromboembolism who were hospitalized in our hospital during the same period were analyzed and compared with group 1. Laboratory tests pertaining to the day of neuroimaging (C-reactive protein (CRP), D-dimer level, lymphocyte count, and lactate dehydrogenase (LDH)) and blood O_2_ saturation, as well as epidemiological data such as age, sex, and underlying diseases of the patients, were retrieved from the hospital database and recorded.

### 2.1. Imaging Technique

In all CT examinations, 128- and 16-slice multidetector spiral CT scanners (Somatom Definition, Siemens Healthcare, Forchheim, Germany; Optima 520, GE Medical System, USA) were used. In unenhanced brain CT analyses, automatic dosing (120 kV/auto-mAs) was achieved on the axial plane from the skull base to the vertex level with slice thicknesses of 5 mm and 1.25 mm. Coronal and sagittal reconstruction of the images was achieved in the bone and soft tissue algorithm at the slice thickness and interval of 1.5–2 mm. Brain-neck CT angiography analyses were performed with automatic dosing (120 kV/auto-mAs) at the slice thickness and interval of 0.625–1.25 mm on the axial plane, and coronal and sagittal reformatted images with slice thicknesses of 1.5–2 mm were generated. MRI of the brain was performed with 1.5 T scanners (Magnetom Avanto, Siemens Healthcare, Germany; Optima 360, GE Medical System, USA). In the MRI of the brain, T1-weighted (T1W), T2-weighted (T2W), fluid-attenuated inversion recovery (FLAIR), diffusion-weighted imaging (DWI), and susceptibility-weighted imaging (SWI) sequences on the axial plane; T2W and FLAIR on the sagittal plane; T2W sequences on the coronal plane; and where necessary, following administration of 0.1 mmol/kg gadolinium, T1W sequences on the coronal, sagittal, and axial planes were obtained.

### 2.2. Statistical Analysis

All statistical analyses were performed using SPSS software ver. 23.0 (SPSS Inc, Chicago IL, USA). The Shapiro-Wilk test was used to test the data for normality of distribution. Categorical variables were presented as frequencies (percentages) and compared with the chi-square test (or Fisher's exact test, where appropriate). Nonnormally distributed continuous variables were presented as median with interquartile range (IQR, 25th and 75th percentiles) and compared with the Mann-Whitney *U* test between the groups. After the possible factors were identified by univariate analyses, a logistic regression was performed to ascertain the effect of age, male gender, hospitalization, presence of hypertension, cardiovascular disease, renal insufficiency, previous cerebrovascular disease, and CRP and D-dimer levels on the likelihood that participants have abnormal brain imaging result. The logistic regression model was statistically significant, *χ*^2^(4) = 38.003, *p* < 0.001. The model fit was assessed using the Hosmer and Lemeshow goodness-of-fit test. The model explained 17.0% (Nagelkerke *R*^2^) of the variance in having abnormal brain imaging result and correctly classified 89.4% of cases. For all comparisons, a value of *p* < 0.05 was considered statistically significant.

## 3. Results

### 3.1. Neuroimaging Use and Common Findings

A total of 436 patients were included in this retrospective study: the mean age was 62.4 (18–95) years, and 245 (56.2%) patients were male. Among all cases, 294 (67.4%) patients had at least one comorbidity, and hypertension was the most common comorbidity found (230 patients, 52.8%). The patients undergoing imaging included 329 (75.5%) hospitalized patients and 107 (24.5%) outpatients due to COVID-19. The most common indications for conducting neuroimaging procedures were altered state of consciousness (247 patients, 56.7%), neurological deficit (74 patients, 17%), and syncope (52 patients, 11.9%) (Table 1). The most common used neuroimaging technique was the CT scan of the brain performed in 423 (97%) patients, which was resorted to for a total of 462 times including repetitions. The mean period of time until neuroimaging analysis was calculated to be 7 days for CT of the brain and 8 days for MRI and DWI. Possible ischemic changes were the most common finding in both brain CT (loss of attenuation in white matter in 135 patients, 31.6%) and brain MRI (foci of increased signal in T2A and FLAIR sequences in white matter in 123 patients, 28.2%). In the comparison between group 1 (*N* = 46) and group 2 (*N* = 390), there was a statistically significant difference in terms of presence of at least one comorbid disease, immunosuppression, and duration of hospitalization (*p* = 0.047, *p* < 0.001, and *p* < 0.001, respectively). No statistically significant difference was found in the comparison of age and sex among groups (*p* = 0.448 and *p* = 0.801, respectively). In the comparison between group 3 (*N* = 189) and group 4 (*N* = 247) with normal neuroimaging findings, there was a statistically significant difference in terms of age, gender, presence of at least one comorbid disease, and neuroimaging indications (*p* < 0.001, *p* < 0.001, *p* < 0.001, and *p* < 0.001, respectively) (Table 1).

### 3.2. Acute Hemorrhage

Intracranial hemorrhage was detected in 8 (1.9%) patients in brain CT examination, and 4/8 (50%) of them were gross hemorrhage and subarachnoid hemorrhage. In MR imaging, intracranial hemorrhage was detected in 10 (2.3%) patients with SWI sequence. Of these, 3/10 (30%) were gross hemorrhage (3 nontraumatic patients with gross hemorrhage on CT) and 7/10 (70%) were microhemorrhages. Amyloid angiopathy and chronic hypertensive encephalopathy are particularly involved in the etiology of microhemorrhage. In our study, there was no case with findings suggestive of amyloid angiopathy, whereas 7 of 10 patients with microhemorrhage had hypertension. Traumatic cerebral hemorrhage was observed in one patient.

### 3.3. Acute/Subacute Infarcts and Encephalitis

**Figure 2:**
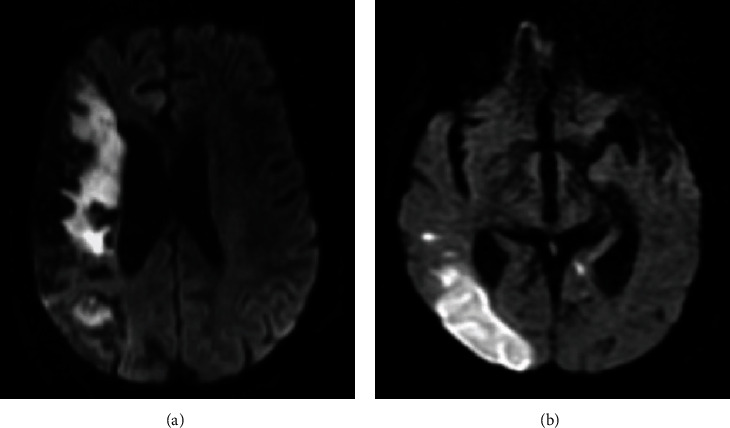
Samples of acute/subacute infarction in 2 different patients (a, b). (a) On DWI, right middle cerebral artery infarction was observed in a 69-year-old female patient affecting most of the right frontal and parietal lobes. (b) The diffusion restriction was observed in DWI due to large infarction in the posterior cerebral artery irrigation area in the right occipital lobe.

**Figure 3:**
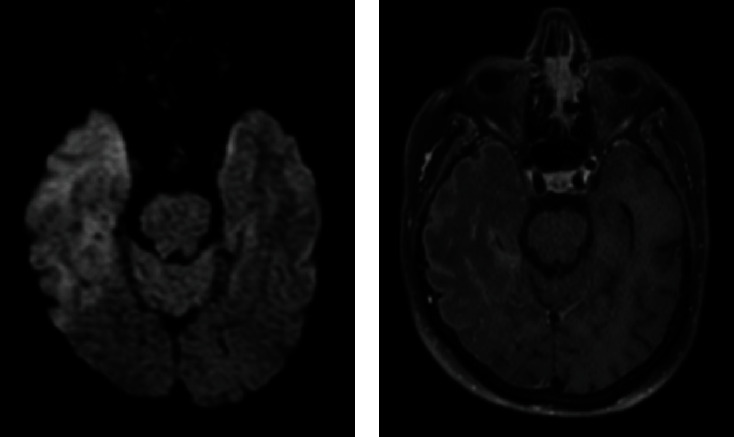
Right temporal lobe predominant encephalitis in a 58-year-old male patient (a, b). (a) Moderate diffusion restriction was observed in the DWI image. (b) In the postcontrast T1-weighted image, moderate enhancement in the subcortical and marked enhancement in the subpial subarachnoid were observed.

On DWI, 46 (28.2%) of 163 patients had findings consistent with infarction, and 35 (76.1%) of them were evaluated in favor of ischemic infarction (Figure 2). In particular, 29 patients with intracranial hemorrhage or infarction were also administered CTA or MRA; of these, 11 (37.9%) had arterial occlusion and one had venous sinus thrombosis. Since encephalitis was suspected in 3 patients in whom neuroimaging was performed due to altered state of consciousness and confusion, cerebrospinal fluid (CSF) analysis was performed by lumbar puncture, and the findings were consistent with encephalitis (Figure 3). A complete summary of different imaging modalities, their findings, and the average time between the diagnosis of COVID-19 and the first radiological examination is given in Table 2.

### 3.4. Laboratory and Other Findings

**Figure 4:**
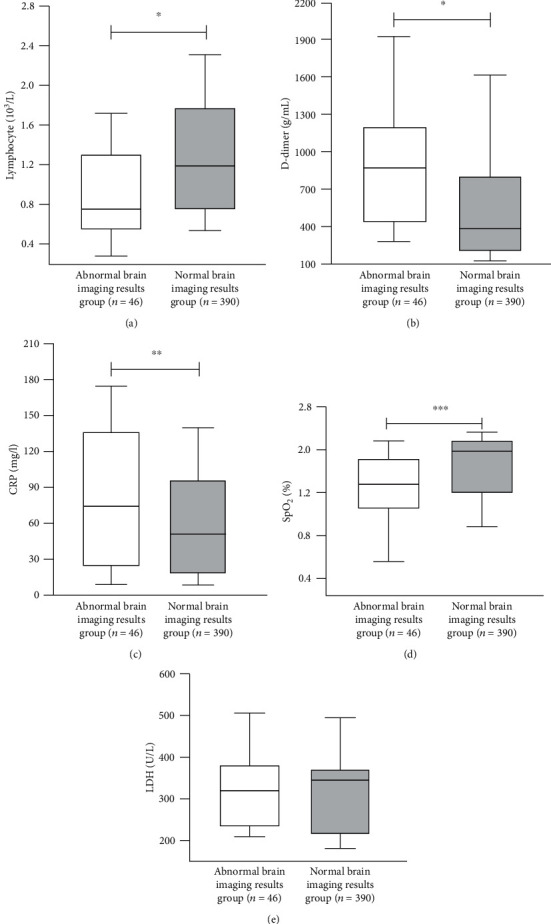
Differences in baseline characteristics of patients according to study groups: (a) lymphocyte, (b) D-dimer, (c) CRP, (d) SpO_2_, and (e) LDH. ^∗^*p* < 0.001, ^∗∗^*p* = 0.014, ^∗∗∗^*p* = 0.002, and *p* = 0.067. Data are presented with the Tukey box-and-whisker plot, where the middle line represents the median and the box represents the IQR (the 25th and 75th percentiles).

**Figure 5:**
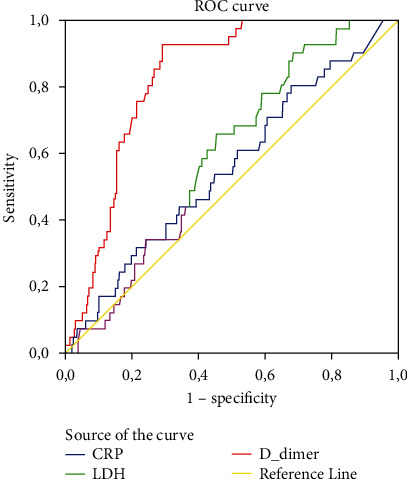
ROC curves for CRP, D-dimer, and LDH in predicting acute/subacute infarction in COVID-19 patients.

Duration of hospitalization and mortality rate were significantly higher in group 1 compared with group 2 (*p* = 0.039 and *p* = 0.014, respectively). Serum levels of CRP and D-dimer were significantly higher in group 1 (*N* = 46) compared to group 2 (*N* = 390), while serum lymphocyte count and SpO_2_ values were significantly lower (*p* = 0.014, *p* < 0.001, *p* < 0.001, and *p* = 0.002, respectively) (Figure 4). In multivariate analysis, there was a 67% decrease in the likelihood of abnormal neuroimaging findings for each unit increase in lymphocyte count (odds ratio (OR) 0.33 (0.16–0.69)). Patients with renal insufficiency were 3.03 times more likely to have an abnormal brain imaging result (Table 3). The optimum cut-off values of serum D-dimer, CRP, and LDH are given in Table 4 to predict acute/subacute infarction. Compared with the non-COVID acute/subacute thromboembolic patient group (*N* = 40), serum D-dimer and CRP levels were found to be significantly higher and lymphocyte counts were significantly lower in COVID-19-positive acute/subacute thromboembolism cases (*p* = 0.02, 0.005, and <0.001, respectively) (Table 5) (Figure 5).

## 4. Discussion

Neuroimaging in patients with COVID-19 is usually performed due to changes in mental status and headache, which may develop secondary to hypoxemia and respiratory distress, as well as neurological deficits, syncope, convulsions, or trauma. The most common cause of neuroimaging was altered consciousness at a rate of 68% (214/329 inpatients) in hospitalized patients and 56.7% in the all study population. The second most common reason for neuroimaging was focal neurological deficit, with a rate of 17% in the cohort and 13.7% (45/329 inpatients) among hospitalized patients. What is striking here is that the incidence of altered state of consciousness was higher in inpatients and that of focal neurological deficit was higher in outpatients compared with in the study cohort. This can be explained by altered mental status that may develop secondary to respiratory distress and low oxygen saturation in blood in hospitalized patients. Radmanesh et al. reported that the 3 most common clinical indications for neuroimaging were altered mental status (42.1%), syncope/fall (32.6%), and focal neurological deficit (12.4%) [15].

The most common abnormal finding in the neuroimaging results of 436 patients included in our study cohort was nonspecific white matter changes evaluated in 189 (43.3%) patients. In the group with nonspecific white matter changes, mean patient age and duration of hospitalization, presence of comorbid disease, and mortality rate were found to be significantly higher than in the group of patients without abnormal neuroimaging findings. One of the reasons for this may be that the rate of presence of accompanying comorbid diseases was significantly higher in these patients compared with the group without abnormal findings [16]. In addition, presence of comorbid diseases and mortality rates were significantly higher in the group of patients with acute/subacute infarction and hemorrhage than in those without abnormal neuroimaging findings. In a study conducted at a single center in the USA which included 242 patients, it was reported that the mortality rate and the likelihood of developing infarction were significantly higher in the group with white matter microangiopathy than in the group without abnormal findings [15].

In this study, 46 patients had acute/subacute infarction, and the overall incidence in the cohort was 10.5%. In a recently published meta-analysis, it was reported that the rate of acute/subacute infarction observed in neuroimaging procedures ranged from 5.4% to 23.3% in patients with COVID-19 [17]. Jain et al. reported the rate of acute/subacute infarction as 7.7% in their cohort of 454 patients [18]. Low molecular weight heparin (LMWH) was used in all patients with COVID-19 who received inpatient treatment in our center, if there were no contraindications. This may have reduced the likelihood of possible thromboembolic events.

The rate of microhemorrhage and/or intracranial hemorrhage in our study was 3.4%, which was lower than the rates reported by Sawlani et al. (9%) and Yoon et al. (6%) [19, 20]. The most important reasons for these differences may be associated with the nonhomogeneity of the selected cohorts (accompanying comorbidity and other demographic characteristics) and the choice of imaging modality. In the meta-analysis published by Choi and Lee, which included 21 articles/case reports, it was reported that the incidences of microhemorrhage, infarction and encephalitis in COVID-19 patients were significantly higher when mainly MRI was used for neuroimaging compared to studies using CT alone or CT-weighted neuroimaging [17].

In the present study, D-dimer level was found to be significantly higher in 46 COVID-19-positive patients with acute thromboembolic events. In addition, in the comparison with the group of 40 cases with non-COVID acute/subacute thromboembolism, it was found that the D-dimer and CRP levels were significantly higher and the lymphocyte count was significantly lower in the COVID-19-positive group. It has been reported that viral infections, and in particular SARS-CoV-2, are associated with an increase in prothrombotic events such as ischemic stroke [21–23]. It has been claimed that viral infections lead to an increase in procoagulant markers, leading to thrombosis as well as disseminated intravascular coagulation and hemorrhagic events [23]. In a study published by Beyrouti et al., significantly high D-dimer levels were observed in all 6 patients who were followed up with the diagnosis of COVID-19 and who experienced acute thromboembolic events during this period [24]. Kremer et al. reported an elevated D-dimer level (>1000 *μ*g/L) in 10 of 11 patients with acute thromboembolic events [25].

Encephalitis cases presenting with quite different rates and radioclinical data caused by SARS-CoV-2 have been reported. In our study, there were clinical, laboratory, and radiological findings consistent with encephalitis in 3 (0.7%) patients. Two of the patients had predominant right frontoparietal and temporal lobe and left frontobasal lobe involvement. Significant increase in signal was observed in T2W and FLAIR sequences, and patchy diffusion restriction was observed in DWI. In postcontrast T1W images, contrast enhancement was nodular in the white matter and linear in the meningeal-pial region. In a recently published meta-analysis, it was reported that the incidence of encephalitis among cohort patients ranged from 1.9% to 4.7% [17]. One patient had transient mild encephalopathy and a well-circumscribed lesion localized in the middle part of the splenium of the corpus callosum on MRI. It was observed that the lesion had marked diffusion limitation and disappeared on the follow-up DWIs, and it was primarily evaluated as a cytotoxic lesion of the corpus callosum (CLOCC).

In conclusion, in this study, we aimed to share the neuroimaging findings of patients who developed neurological symptoms during their follow-up due to COVID-19 and to reveal the relationship between acute thromboembolic events and some laboratory parameters. Patients with COVID-19-positive acute/subacute infarcts have significantly higher D-dimer levels compared to patients with COVID-19-negative acute/subacute infarcts. This supports that viral infections in general, and SARS-CoV-2 in particular, increase the risk of acute thromboembolism by increasing procoagulant markers (inflammatory or autoimmune). We believe that the risk of acute thromboembolism increases significantly in COVID-19 patients when serum D-dimer level is above 756 ng/dL, and detailed neurological evaluation and neuroimaging when necessary in these patients will be beneficial.

First of all, since CT was used predominantly in our study, some pathologies that could be detected by MR, and especially the changes in the cortex or white matter, might not have been detected. In addition, the necessary neuroimaging could not be performed in some of the patients who were being treated under intensive care conditions due to the failure to provide appropriate technical requirements.

## Figures and Tables

**Table 1:** Comparison of characteristics of patients with and without abnormal brain imaging result.

Variables	Overall (*n* = 436)	Abnormal brain imaging result group (*n* = 46)^a^	Normal brain imaging result group (*n* = 390)^b^	*p* value	Abnormal brain imaging result group (*n* = 189)^c^	Normal brain imaging result group (*n* = 247)^d^	*p* value
Age (years)	67 (54-76)	69.5 (53-79)	66.0 (54-75)	0.448	71.0 (60-79)	63.0 (44-73)	<0.001^∗^
Gender, male	245 (56.2)	32 (69.6)	213 (54.6)	0.053	129 (68.3)	116 (47.0)	<0.001^∗^
Hospitalization	329 (75.5)	42 (91.3)	287 (73.6)	0.008^∗^	160 (84.7)	169 (68.4)	<0.001^∗^
Any comorbidity	294 (67.4)	37 (80.4)	257 (65.9)	0.047^∗^	146 (77.2)	148 (59.9)	<0.001^∗^
Hypertension	230 (52.8)	29 (63.0)	201 (51.5)	0.139	113 (59.8)	117 (47.4)	0.010^∗^
Previous cerebrovascular disease	59 (13.5)	9 (19.6)	50 (12.8)	0.206	38 (20.1)	21 (8.5)	<0.001^∗^
Cardiovascular disease	89 (20.4)	11 (23.9)	78 (20.0)	0.533	47 (24.9)	42 (17.0)	0.044^∗^
Renal insufficiency	32 (7.3)	8 (17.4)	24 (6.2)	0.012^∗^	21 (11.1)	11 (4.5)	0.008^∗^
Diabetes mellitus	93 (21.3)	12 (26.1)	81 (20.8)	0.405	44 (23.3)	49 (19.8)	0.385
COPD	38 (8.7)	1 (2.2)	37 (9.5)	0.160	15 (7.9)	23 (9.3)	0.614
Any immunosuppression	10 (2.2)	5 (10.8)	5 (1.3)	<0.001^∗^	7 (3.7)	3 (1.2)	0.656
Indications for brain imaging	—	—	—	0.239	—	—	<0.001^∗^
Altered mental status	247 (56.7)	28 (60.9)	219 (56.2)		123 (65.1)	124 (50.2)	
Syncope	52 (11.9)	3 (6.5)	49 (12.6)		10 (5.3)	42 (17.0)	
Neurological deficit	74 (17.0)	13 (28.3)	61 (15.6)		39 (20.6)	35 (14.2)	
Seizure	9 (2.1)	0 (0)	9 (2.3)		2 (1.1)	7 (2.8)	
Head trauma	12 (2.8)	0 (0)	12 (3.1)		3 (1.6)	9 (3.6)	

^∗^Statistically significant. Values are presented as median (interquartile range) for continuous variables and frequency (percentage) for categorical variables. COPD: chronic obstructive pulmonary disease. ^a^Group 1, ^b^Group 2, ^c^Group 3, and ^d^Group 4.

**Table 2:** Brain imaging results of 436 COVID-19 patients.

Variable	Value
Brain CT (*n* = 423)	
Time^∗^ of first CT exam (days)	7 (6-9)
No abnormality	265 (62.7)
Infarction	15 (3.5)
Intracranial hemorrhage	8 (1.9)
Nonspecific white matter changes	135 (31.9)
Brain MRI (*n* = 243)	
Time^∗^ of first MR exam (days)	8 (6-10)
Intracranial hemorrhage (for SWI)	10 (4.1)
Nonspecific white matter changes	123 (50.6)
Location of nonspecific white matter changes (*n* = 123)	
Frontal lobe	78 (63.4)
Parietal lobe	68 (55.3)
Temporal lobe	32 (26.0)
Occipital lobe	25 (20.3)
Pons-mesencephalon	19 (15.4)
Basal ganglia	32 (26.0)
Corpus callosum	12 (9.8)
Periventricular	41 (33.3)
Cerebellar	7 (5.7)
CTA (*n* = 18)	
Occlusion	8 (44.4)
Right MCA	5 (62.5)
Left MCA	1 (12.5)
Left PCA	2 (25.0)
MRA (*n* = 11)	
Occlusion	5 (45.5)
Right MCA	3 (60.0)
Right PCA	1 (20.0)
Venous sinus thrombosis	1 (20.0)
DWI (*n* = 163)	
Time^∗^ of first diffusion exam (days)	8 (6-10)
Infarction	46 (28.2)
Infarction site	
Right hemisphere	13 (28.3)
Left hemisphere	12 (26.1)
Both hemisphere	18 (39.1)
Cerebellum	3 (6.5)

^∗^Interval between diagnosis of COVID-19 and first brain imaging exam.

**Table 3:** Results of multivariate logistic regression model in predicting the presence of acute/subacute thromboembolism in COVID-19 patients.

Variable	OR	95% CI	*p* value
Age	0.981	0.960-1.002	0.083
Male gender	1.673	0.802-3.492	0.170
COPD	0.169	0.022-1.322	0.090
Diabetes mellitus	1.986	0.824-3.542	0.079
Hypertension	1.943	0.938-4.026	0.074
Renal insufficiency	3.030	1.108-8.292	0.031^∗^
CRP	0.997	0.993-1.002	0.289
D-dimer	1.000	0.984-1.003	0.002^∗^
LDH	1.004	0998-1.007	0.341
Lymphocyte	0.336	0.162-0.696	0.003^∗^
SpO_2_	0.941	0.885-1.002	0.056

^∗^Statistically significant. OR: odds ratio; Cl: confidence interval; CRP: C-reactive protein; COPD: chronic obstructive pulmonary disease.

**Table 4:** Comparison of area under curve (AUC) to predict the acute/subacute thromboembolism in COVID-19 patients.

	AUC (95% CI)	Cut-off	Sensitivity	Specificity	*p* value
CRP (mg/L)	0.557 (0.465-0.648)	8.4	0.926	0.691	<0.230
D-dimer (ng/mL)	0.828 (0.782-0.875)	756	0.829	0.739	<0.001
LDH (U/L)	0.595 (0.519-0.672)	0.56	0.667	0.392	0.045

CRP: C-reactive protein; LDH: lactate dehydrogenase.

**Table 5:** Demographic and laboratory data in acute/subacute thromboembolism cases.

Variables	COVID-19-positive group (*n* = 46)	Non-COVID group (*n* = 40)	*p* value
Age (years)	61.1 (51.0-79.0)	66.2 (54.0-86.0)	0.215
Gender, male (%)	32 (69.6)	12 (30.0)	0.003^∗^
Any comorbidity (%)	37 (80.4)	31 (77.5)	0.323
Mortality (%)	14 (30.4)	7 (17.5)	0.194
CRP level (mg/L)	78.7	31.7	0.005^∗^
LDH level (U/L)	362.6	306.2	0.098
D-dimer level (ng/mL)	2506.8	631.8	0.020^∗^
Lymphocyte (10^3^/*μ*L)	1.23	2.55	<0.001^∗^

^∗^Statistically significant. ^a^The most common reason. CRP: C-reactive protein; LDH: lactate dehydrogenase.

## Data Availability

The data used to support the findings of this study are available from the corresponding author upon request.
